# The effects of foliar fertilization with iron sulfate in chlorotic leaves are limited to the treated area. A study with peach trees (*Prunus persica* L. Batsch) grown in the field and sugar beet (*Beta vulgaris* L.) grown in hydroponics

**DOI:** 10.3389/fpls.2014.00002

**Published:** 2014-01-20

**Authors:** Hamdi El-Jendoubi, Saúl Vázquez, Ángeles Calatayud, Primož Vavpetič, Katarina Vogel-Mikuš, Primož Pelicon, Javier Abadía, Anunciación Abadía, Fermín Morales

**Affiliations:** ^1^Department of Plant Nutrition, Aula Dei Experimental Station (CSIC)Zaragoza, Spain; ^2^Departamento de Horticultura, Instituto Valenciano de Investigaciones AgrariasValencia, Spain; ^3^Department of Low and Medium Energy Physics, Jožef Stefan InstituteLjubljana, Slovenia; ^4^Department of Biology, Biotechnical Faculty, University of LjubljanaLjubljana, Slovenia

**Keywords:** *Prunus persica*, *Beta vulgaris*, foliar Fe nutrition, leaf anatomy, leaf chlorosis, leaf Fe localization

## Abstract

Crop Fe deficiency is a worldwide problem. The aim of this study was to assess the effects of foliar Fe applications in two species grown in different environments: peach (*Prunus persica* L. Batsch) trees grown in the field and sugar beet (*Beta vulgaris* L. cv. “Orbis”) grown in hydroponics. The distal half of Fe-deficient, chlorotic leaves was treated with Fe sulfate by dipping and using a brush in peach trees and sugar beet plants, respectively. The re-greening of the distal (Fe-treated) and basal (untreated) leaf areas was monitored, and the nutrient and photosynthetic pigment composition of the two areas were also determined. Leaves were also studied using chlorophyll fluorescence imaging, low temperature-scanning electron microscopy microanalysis, scanning transmission ion microscopy-particle induced X-ray emission and Perls Fe staining. The distal, Fe-treated leaf parts of both species showed a significant increase in Fe concentrations (across the whole leaf volume) and marked re-greening, with significant increases in the concentrations of all photosynthetic pigments, as well as decreases in de-epoxidation of xanthophyll cycle carotenoids and increases in photochemical efficiency. In the basal, untreated leaf parts, Fe concentrations increased slightly, but little re-greening occurred. No changes in the concentrations of other nutrients were found. Foliar Fe fertilization was effective in re-greening treated leaf areas both in peach trees and sugar beet plants. Results indicate that the effects of foliar Fe-sulfate fertilization in Fe-deficient, chlorotic leaves were minor outside the leaf surface treated, indicating that Fe mobility within the leaf is a major constraint for full fertilizer effectiveness in crops where Fe-deficiency is established and leaf chlorosis occurs.

## Introduction

Iron deficiency (Fe chlorosis) is a disorder affecting crops in many areas of the world, mainly associated with high pH, calcareous soils that make soil Fe unavailable for plants (Abadía et al., 2011). Iron deficiency has a large economical impact, because crop quality and yield can be severely compromised (Álvarez-Fernández et al., 2011; El-Jendoubi et al., 2011). In the case of high value fruit tree crops, the prevention or correction of Fe chlorosis is usually made by applying expensive fertilizers such as synthetic Fe(III) chelates, in spite of the progress regarding adequate rootstocks tolerant to Fe chlorosis (Lucena, 2006; Rombolà and Tagliavini, 2006).

Iron-deficient plants progressively develop a yellow leaf color, the so-called “leaf chlorosis.” Iron fertilization with a variety of Fe compounds leads to leaf re-greening as well as to a series of biochemical and metabolic changes in leaves and roots. The major sink for Fe is the chloroplast, where the thylakoids and the stromal machinery need large amounts of Fe (Abadía et al., 2011). Many of the studies on the physiological effects of Fe re-supply to Fe-deficient plants described changes observed after Fe is applied to the nutrient solution in plants grown in hydroponics (López-Millán et al., 2001a,b; Larbi et al., 2004, 2010; Jiménez et al., 2009) or after solid implants of Fe compounds were placed in the branches of fruit trees grown in the field (Larbi et al., 2003). Plant species investigated so far include sugar beet (López-Millán et al., 2001a,b; Larbi et al., 2004, 2010), pear and peach (Larbi et al., 2003), and peach-almond hybrid (Jiménez et al., 2009), with physiological responses at the root and leaf levels being described in the different studies.

Iron canopy fertilization (foliar fertilization) can be a cheaper, more environmentally-friendly alternative to soil treatments with synthetic Fe(III) chelates for the control of Fe chlorosis in fruit trees (Pestana et al., 2003; Álvarez-Fernández et al., 2004; Fernández et al., 2013). Foliar fertilization with Fe is traditionally used in crops where the use of chelates is too expensive (Wójcik, 2004). The success of foliar treatments with Fe-containing formulations depends on many factors, including the capacity to penetrate the cuticle and/or stomata, undergo transport through the apoplast and cross the plasma membrane of leaf cells to reach the cytoplasm and then the chloroplast (Rombolà et al., 2000; Fernández et al., 2009, 2013; Abadía et al., 2011). Iron(II)-sulfate has been tested as a foliar fertilizer in several fruit crop studies, and increases in leaf chlorophyll (Chl) concentrations in kiwi (Rombolà et al., 2000), citrus (Pestana et al., 2001, 2003), pear (Álvarez-Fernández et al., 2004), grapevine (Yunta et al., 2013), and peach trees (Fernández et al., 2006, 2008) have been reported. Foliar Fe fertilization could also improve fruit size and quality, as observed in *Citrus* species (El-Kassa, 1984; Pestana et al., 1999, 2001). In a study assessing the effectiveness of foliar applications of FeSO_4_ to re-green chlorotic pear trees, it was concluded that foliar fertilization cannot be considered as good alternative for full control of Fe chlorosis, but could be used instead as a complementary technique to soil Fe(III) chelate application (Álvarez-Fernández et al., 2004). Although foliar Fe fertilization seems to be potentially effective, the scientific background for this practice is still scarce (Abadía et al., 1992, 2011; Rodríguez-Lucena et al., 2010; Fernández et al., 2013), and little is known on the mobility of the leaf surface-applied Fe, both across the leaf volume and to adjacent leaf areas.

In contrast with fruit trees, where foliar Fe fertilization is generally used in chlorotic leaves, canopy Fe-fertilization is increasingly being used in cereal crops to increase the Fe concentration in grains, in what is called biofortification. In these crops, which are generally treated with foliar Fe sprays when there is no leaf chlorosis, applied Fe has been shown to re-translocate efficiently to other plant organs, both in wheat (Cakmak et al., 2010; Zhang et al., 2010; Aciksoz et al., 2011) and rice (Wei et al., 2012; He et al., 2013). The possible role of senescence processes, known to facilitate Fe re-translocation within the plant (Zhang et al., 1995; Shi et al., 2012), in the redistribution of the Fe applied in foliar fertilizers has not been explored yet.

In this study, we have used an array of techniques to investigate the effects of Fe applied as Fe-sulfate to Fe-deficient leaves, by looking at treated and untreated leaf surfaces. An Fe-containing formulation was applied only to the distal half of leaves from peach trees grown in the field and from sugar beet plants grown in hydroponics. The fertilizer solution consisted in 2 mM FeSO_4_ supplemented with a surfactant, a formulation that has been found to have a good re-greening effect in previous studies (Fernández et al., 2006, 2008; El-Jendoubi et al., 2011). The effects of Fe fertilization in treated and untreated leaf areas were assessed from changes in SPAD and the total concentrations of Fe and photosynthetic pigments. Chlorophyll fluorescence imaging was also used to assess differential changes in treated and untreated leaf areas. Furthermore, the distribution of Fe in leaf transversal sections was studied using three different image techniques: optical microscopy (Perls-DAB staining, reflecting labile Fe pools), low temperature scanning electron microscopy coupled to microanalysis (LT-SEM-EDX, providing fine leaf structure and a semi-quantitative Fe measurement), and scanning transmission ion microscopy-particle induced X-ray emission (STIM μPIXE, giving a quantitative Fe measurement).

## Materials and methods

### Field growth conditions for peach trees

**Figure 1:**
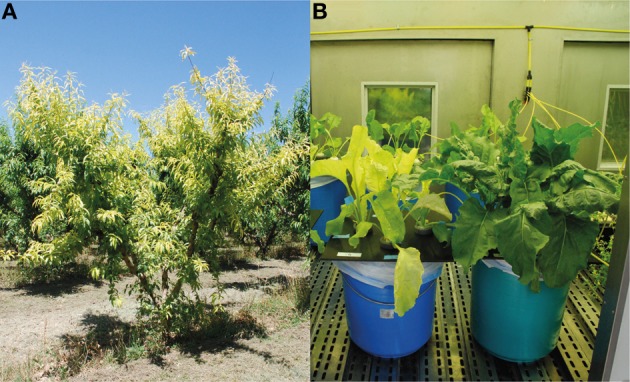
**(A)** Iron-deficient peach trees grown in the field. **(B)** Sugar beet plants grown in hydroponics. Sugar beet plants were grown in Fe-deficient (left side) or Fe-sufficient (right side) conditions.

A peach tree (*Prunus persica* L. Batsch) orchard was selected near the village of Plasencia de Jalón (Zaragoza province), in the Ebro river valley in North-Eastern Spain (41°40′27.72″N, 1°13′33.46″O). The orchard was on a calcareous soil (*Typical xerofluvent*, clay-loamy texture), with 30% total CaCO_3_, 10% active CaCO_3_, 7 mg kg^−1^ DTPA-extractable Fe, 2.6% organic matter and pH 7.8 in water. Trees were of the cv. “Miraflores” grafted on GF677 rootstock, 16-year old and with a frame 5 × 4 m. Trees were flood-irrigated approximately every 2–3 weeks. This orchard developed Fe chlorosis as many others in the area (Figure 1A). Normal fertilization practices were used, with the exception of Fe fertilization, which was totally excluded from the grower treatments in the selected trees.

### Hydroponic growth conditions for sugar beet plants

Sugar beet (*Beta vulgaris* L. cv. “Orbis”) plants were grown in a controlled environment chamber with a photosynthetic photon flux density (PPFD) of 350 μmol m^−2^ s^−1^ at leaf height, and a 16 h-22°C/8 h-19°C day/night regime. Seeds were germinated and grown in vermiculite for 2 weeks. Seedlings were grown for 3 more weeks in half-strength Hoagland nutrient solution with 45 μM Fe(III)-EDTA [Fe(III)-ethylenediaminetetraacetate]. Then, seedlings were transferred to 20 L plastic buckets containing half-strength Hoagland nutrient solution with either 0 (−Fe) or 45 μM Fe(III)-EDTA (+Fe, Fe-sufficient control plants; pH 5.5). The pH of the Fe-free nutrient solutions was buffered at approximately 7.7 by adding 1 mM NaOH and 1 g L^−1^ of CaCO_3_, a treatment that simulates conditions usually found in the soils associated with Fe deficiency (Susín et al., 1996). After growing for 14 days under these conditions, plants grown in the zero Fe treatment showed Fe-deficiency symptoms, including leaf chlorosis (Figure 1B).

### Foliar iron treatments for peach trees

**Figure 2:**
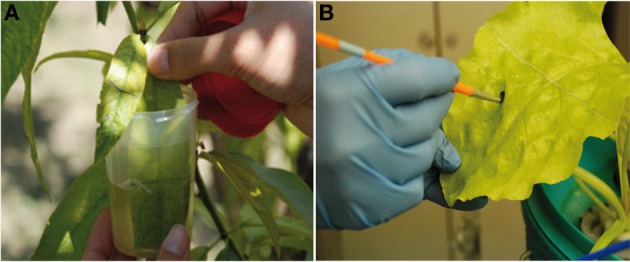
**Treatment of the distal half part of (A) peach tree leaves grown in the field by dipping into the Fe formulation and (B) sugar beet leaves grown in hydroponics using a paintbrush to apply the Fe formulation**. In both cases, a solution containing 2 mM FeSO_4_ and 0.1% surfactant was used.

Field treatments in peach trees were made in 3 consecutive years in the same orchard, according with fertilization practices indicated in El-Jendoubi et al. (2011). Iron-deficient peach trees with a similar leaf chlorosis level were chosen in early June each year. These Fe-deficient trees were not treated with Fe at the beginning of the growing season. Before treatment, all Fe-deficient trees had SPAD values of approximately 18 ± 2 (115–151 μmol Chl m^−2^), indicative of Fe chlorosis, whereas Fe-sufficient trees had SPAD values of approximately 31–35 (250–291 μmol Chl m^−2^). In mid-June, 40 similar shoots per tree were selected in each of four different Fe-deficient trees. From these, 20 shoots were fertilized with Fe (only the distal half of the leaf, see below) whereas the other 20 were kept as Fe-deficient, not fertilized controls. Leaves at the positions 4^th^–5^th^ from the top (young and fully developed) in each shoot were labeled with color tape, and the distal half part of the labeled leaves was immersed briefly (for approximately 2 s) in a solution containing 2 mM FeSO_4_ and 0.1% BreakThrough S-233 (a non-ionic, organo-silicon surfactant; polyether- modified polysiloxane, from Evonik Industries AG, Essen, Germany) (Figure 2A). The fertilizer was applied from 8:00 to 10:00 h solar time, and temperature and relative humidity during the treatments were approximately 18–20°C and 60–80%, respectively. The solution pH was 4.0 and the formulation was applied immediately after preparation to minimize atmospheric Fe oxidation (Fernández et al., 2006). A second application with the same formulation was made 4 weeks later. The experiment was carried out thrice, in the summers of 2009, 2010, and 2011. In 2009, only the assessment of re-greening effects and the analysis of mineral elements were carried out, whereas in 2010 and 2011 all parameters were measured. To carry out soil Fe-chelate (Fe(III)-EDDHA) application, five wells (approximately 20 cm-deep, 20 × 20 cm-wide) were excavated in the soil around each tree in mid-June, approximately 100 cm from the trunk, and 10 g of Fe(III)-EDDHA (Sequestrene 138 from Syngenta; 6.2% chelated Fe) was placed in the uncovered soil surface of each well (this corresponds to a dose of approximately 3 g of Fe per tree). The wells were topped again with soil and 4 L of water per well were added.

### Foliar iron treatments for sugar beet plants

A solution containing 2 mM FeSO_4_ and 0.1% BreakThrough S-233 was applied to the distal half part of leaves, on both the adaxial and abaxial leaf sides, using a paintbrush (Figure 2B). The application was made approximately 4 h after the onset of the illumination in the chamber. The application was made twice, the first one at the beginning of the experiment and the second 2 days later. The experiment was carried out twice, each time with a different batch of plants.

### Assessment of leaf re-greening after foliar fertilization

In peach trees, the assessment of the leaf re-greening was carried out weekly, by measuring the leaf Chl concentration in the 40 labeled shoots (20 treated and 20 untreated with Fe) in each of the four trees. Leaf Chl was estimated in every leaf using a SPAD 502 meter (Minolta Co., Osaka, Japan), measuring in the midst of the distal treated and basal untreated areas (two measurements each). In the non-fertilized, control leaves, measurements were also made in the distal and basal leaf parts. Values shown are means ± SE (*n* = 4 trees, using the averages of the 20 leaves measured in each tree). The time course of the changes in the SPAD values was expressed as the relative increment at each measurement time compared to the initial values before the first application.

In sugar beet, the re-greening effect was assessed daily by estimating the leaf Chl concentration with the SPAD device in four different leaves per plant. In each leaf, four measurements were made in the distal treated area and four more in the basal untreated area. In the unfertilized control leaves, measurements were also made in the distal and basal leaf parts. Values shown are means ± SE (*n* = 4 plants, using the average of 4 leaves per plant). The time course of the changes in the SPAD values was expressed as relative increments with respect to the initial values, as indicated above for peach leaves.

### Leaf mineral analysis

At the end of the experimental period (8 weeks after the first application in peach trees and 7 days after the first application in sugar beet), leaves were excised and the mineral element concentrations of the distal treated and basal untreated areas were analyzed according to standard laboratory procedures (Belkhodja et al., 1998b; Igartua et al., 2000). Each leaf was divided in two parts, treated and untreated, discarding a 5-mm strip in the border zone. Prior to processing, both leaf sides were washed, first with 0.1% detergent (Mistol, Henkel) solution to remove surface contamination, then with tap water and finally with ultrapure water. Results were expressed as % of dry weight (DW) for macronutrients (N, P, K, Ca, and Mg) and as μg g^−1^ DW for micronutrients (Fe, Cu, Mn, and Zn).

### Photosynthetic pigment determination

At the end of the experimental period, four disks per leaf part and treatment were taken with a calibrated cork borer from peach trees and sugar beet plants. Disks were wrapped in aluminum foil, frozen in liquid N_2_ and taken to the laboratory to be stored (still wrapped in foil) at −20°C. Leaf pigments were extracted with acetone in the presence of Na ascorbate and stored as described previously (Abadía and Abadía, 1993). Pigment extracts were thawed on ice, filtered through a 0.45 μm filter and analyzed by HPLC (Waters 600 pump and 996 photodiode array detector) (Larbi et al., 2004). Pigments determined were Chl *a*, Chl *b*, neoxanthin, lutein, β-carotene, violaxanthin, antheraxanthin, and zeaxanthin. All chemicals used were HPLC quality, and the analysis time for each sample was 15 min.

### Low temperature-scanning electron microscopy and microanalysis (LT SEM-EDX) of transversal leaf sections

At the end of the experimental period, images of cryo-fractured transversal peach tree leaf sections were obtained with a digital scanning electron microscope (SEM) (Zeiss DSM 960, Oberkochen, Germany) as described elsewhere (Ojeda-Barrios et al., 2012). Sections of fresh peach leaf tissue (2.5 × 2.5 mm leaf pieces) were mounted on aluminum stubs, cryo-fixed in slush N_2_, cryo-transferred to a vacuum chamber at −180°C, and fractured using a stainless steel spike. Once inside the microscope, the samples underwent superficial etching under vacuum and were overlaid with gold. Fractured samples were observed at low temperature using secondary and back-scattered (BSE) electrons.

Semi-quantitative Fe analysis in the peach tree transversal leaf sections was carried out using microprobe analysis with an Energy Dispersive X-ray (EDX) system (Pentaflet, Oxford, UK), using only smooth surfaces (Hess et al., 1975). Semi-quantitative analysis was carried out using standard ZAF (atomic number, absorption and fluorescence) correction procedures with Link Isis (Oxford, UK) v.3.2 software. Eight points of analysis per leaf tissue and three leaves per treatment were analyzed.

### Scanning transmission and ion microscopy-particle induced X-ray emission (STIM μPIXE) analysis of transversal leaf sections

At the end of the experimental period, quantitative STIM-μPIXE elemental microanalysis was carried out in peach tree leaves. Sample preparation and microanalysis conditions were as described elsewhere (Vogel-Mikuš et al., 2009, 2010). Selected leaf areas of the main vein and its closest lamina area were sectioned with a razor blade in small pieces (2 × 5 mm), inserted in a 2 mm stainless steel needle, and dipped into liquid propane cooled by liquid N_2_. Leaf pieces were cross-sectioned (30 μm) with a Leica (Bensheim, Germany) CM3050 cryotome at −25°C. Leaf sections were placed in Al holders and freeze-dried at −50°C at 0.04 mbar for 3 d. Dry sections were mounted on an Al holder between two thin layers of Pioloform foil (SPI supplies, West Chester, PA, USA). Leaf sections with the best-preserved morphology were measured using μPIXE. High- and low-current modes were applied sequentially at the same sample region of interest with the nuclear microprobe. In the high-current mode used for μPIXE analysis, a proton beam (3 MeV) with a diameter varying from 1 to 3 mm at ion currents ranging from 40 to 500 pA was formed, depending on the required lateral resolution. In the low energy mode, STIM images were used for determination of thickness. μPIXE spectra were collected by a high-purity germanium (HPGe) X-ray detector with an active area of 95 mm^2^, a 25-mm-thick Be window and an energy resolution of 170 eV at 5.9 keV. The HPGe detector was positioned at an angle of 135° with respect to the beam direction and was additionally equipped with a 100-mm thick polyimide absorber to suppress large count rates at X-ray energies below 4 keV. Samples were sprayed with low-energy electrons from a hot tungsten filament to prevent sample charging. The regions of interest on the samples were pre-selected by a short PIXE mapping in a high-current mode, and then PIXE and STIM mapping were recorded in the list mode over a period of at least 10 h. The X-ray and STIM spectra corresponding to distinct morphological structures (upper/lower epidermis, palisade/spongy mesophyll, vascular bundles, etc.) were identified and extracted from the selected regions on the basis of STIM and elemental maps. Assuming a cellulose matrix, the average thickness of the selected area was calculated from the STIM spectrum and used for matrix corrections in the GUPIX program (University of Guelph, Ontario, Canada) used for fitting the PIXE spectra. Finally, the tissue elemental concentrations and elemental maps of selected leaf areas were obtained. The calibration was verified using multi-elemental standard reference materials (Vogel-Mikuš et al., 2009, 2010).

### Perls-diaminobenzidine iron staining of transversal leaf sections

At the end of the experimental period, labile Fe forms in transversal leaf sections were assessed in peach tree leaves using Perls diaminobenzidine (DAB) staining as described elsewhere (Roschzttardtz et al., 2009). Representative areas (25 mm^2^) from the midst of leaf areas adjacent to main veins were embedded in 5% agar and sectioned transversally (70 μm thickness) using a vibrating blade microtome (VT1000 S, Leica Microsystems GmbH, Wetzlar, Germany). Fresh sections were incubated with a 4% K_4_[Fe(CN)_6_], 4% HCl solution for 30 min at room temperature (RT) and 100% RH. Negative controls were run by incubating fresh sections with 4% HCl. After three washes with deionized water, a second incubation with methanol containing 0.01 M NaN_3_ and 0.3% H_2_O_2_ was carried out for 1 h at RT. Sections were washed three times with 0.1 M phosphate buffer pH 7.4 and then incubated with the same buffer containing 0.025% DAB, 0.005% H_2_O_2_, and 0.005% CoCl_2_ for 30 min at RT. Finally, sections were washed with ultrapure water and bright light images (2592 × 1994 pixels) were taken using an inverted microscope (DM IL LED, Leica) with a charge-coupled device (CCD) camera (Leica DFC 240C).

### Chlorophyll fluorescence imaging of leaves

One week after the first foliar application, peach tree leaves were used to investigate the spatial heterogeneity of Chl fluorescence parameters with an imaging-pulse amplitude modulation (PAM) fluorometer (Walz, Effeltrich, Germany) as described elsewhere (Calatayud et al., 2006). A good homogeneity of the actinic light intensity was obtained in the whole illuminated leaf area, and the CCD camera had a resolution of 640 × 480 pixels. Pixel value images of the fluorescence parameters were displayed with help of a false color code, ranging from black through red, yellow, green, blue to pink (from 0.000 to 1.000) (Calatayud et al., 2006). Leaves were kept in the dark for 30 min prior to measurement and for 5 min between measurements. The minimum (F_O_) and maximum fluorescence (F_M_) were obtained by applying measuring light pulses at low frequency (1 Hz) and by applying a saturating blue light pulse (10 Hz), respectively. Fluorescence parameters are according to standard nomenclature (Larbi et al., 2006). Dark-adapted, maximum potential photosystem II (PSII) efficiency was calculated as F_V_/F_M_, where F_V_ is F_M_-F_O_ (Morales et al., 1991; Abadía et al., 1999). Then, actinic illumination was switched on and saturating pulses were applied at 20 s intervals for 5 min in order to determine the maximum fluorescence yield during saturating pulses (F_M_′), and the Chl fluorescence yield during actinic illumination (F_S_). For each interval, saturation pulse images and values of various Chl fluorescence parameters were captured. Actual (Φ_PSII_) PSII efficiency, photochemical (qP) and non-photochemical quenching (NPQ) were calculated as (F_M_′-F_S_)/F_M_′ (Genty et al., 1989), (F_M_′-F_S_)/F_V_′ (Larbi et al., 2006), and (F_M_/F_M_′)-1 (Bilger and Björkman, 1990), respectively.

### Statistical analyses

In all cases, One-Way analyses of variance (ANOVA) were run using the GLM procedure of the SAS package (SAS Institute Inc., 1989) with the exception of EDX (see below). A *post hoc* comparison of means with Duncan's test (*p* ≤ 0.05) was carried out. In the case of the nutrient concentrations, an additional statistical analysis was made using “years” as a fixed factor with “trees” nested into years; then, specific contrasts were carried out to compare the Fe-fertilized vs. the non-fertilized basal parts and the Fe-fertilized vs. the non-fertilized distal parts. In the case of EDX, One-Way ANOVA was used to compare the results obtained in the different leaf tissues (adaxial epidermis, palisade parenchyma, xylem vessels, spongy parenchyma, and abaxial epidermis) using SPSS v.17.0 software. In the case of μPIXE, the microanalysis is a quantification of selected areas, and therefore there were no replicates in order to make statistics.

## Results

### Foliar fertilization with iron sulfate leads to leaf re-greening in peach tree and sugar beet leaves

**Figure 3:**
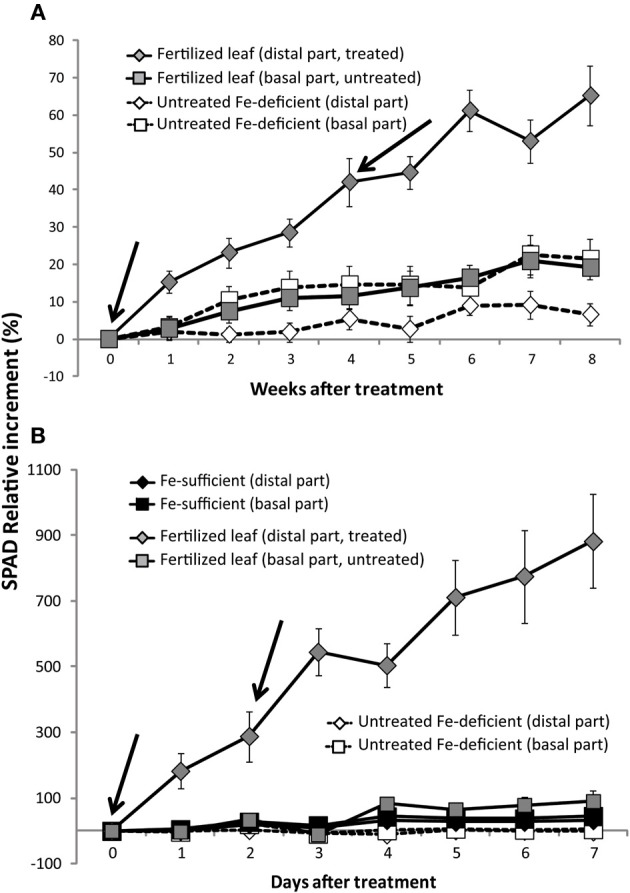
**Time course of the relative changes in leaf SPAD values in peach tree (A) and sugar beet leaves (B)**. The treatment was carried out with a solution containing 2 mM FeSO_4_ and 0.1% surfactant. In peach leaves, foliar treatments were made at weeks 0 and 4, and the SPAD index was measured each week. In sugar beet leaves, the treatment was made at days 0 and 2 and the SPAD index was measured daily. Peach tree data are means ± SE (*n* = 11 trees: 3 in 2009, 4 in 2010, and 4 in 2011; each sample was composed of 20 leaves, each from a different shoot from the same tree; two measurements were taken per half-leaf). Initial SPAD values in chlorotic peach tree leaves were 18 ± 2. Sugar beet data are means ± SE (*n* = 8 plants, 4 in each of two different batches; each sample was composed of four leaves from the same plant; four measurements were taken per half-leaf). Initial SPAD values in sugar beet chlorotic leaves were 11.5 ± 1.5.

**Figure 4:**
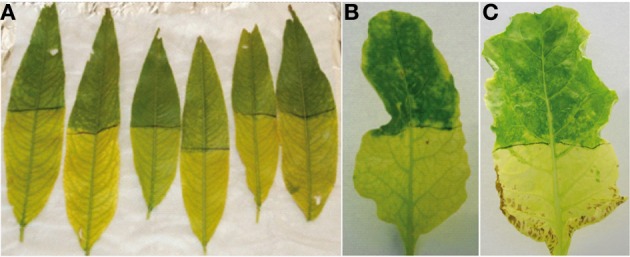
**Images of peach tree leaves 8 weeks after the first foliar Fe treatment (A) and two different sugar beet leaves 7 days after the first treatment (B,C)**. The re-greened areas are the result of treatments with a solution containing 2 mM FeSO_4_ and 0.1% surfactant.

Re-greening of the Fe-treated distal part of Fe-deficient peach tree leaves was already observed 1 week after the first treatment. The increase in SPAD was approximately 16% over the untreated controls (Figure 3A). The re-greening continued in the following weeks and also after the second treatment, which was applied at week 4. At the end of the experiment, 8 weeks after the first Fe treatment, the treated leaf area had a 65% relative increase in SPAD when compared to those of the basal untreated parts of the same leaves (Figure 3A). The same Fe formulation (combination of Fe compound and surfactant) caused a smaller (1.2-fold) relative increase in leaf Chl in a previous study (Fernández et al., 2008). However, in the present work re-greening did not extend into the untreated areas (Figure 4A), in contrast to what was indicated with different Fe fertilizer formulations in a previous study with peach trees (Fernández et al., 2008). The basal leaf parts showed a slight re-greening (increases were always ≤20% when compared to the initial SPAD values; Figure 3A), whereas the re-greening was only minor in the distal part of the untreated leaves.

In the case of sugar beet leaves, leaf re-greening was observed one day after foliar Fe fertilization; the increase was approximately 2-fold (Figure 3B). At the end of the experiment, the treated distal areas had a 9-fold SPAD increase with respect to the initial values. However, the re-greening of the leaf surface was not homogenous (Figure 4B). The untreated basal part of treated leaves and both parts of the untreated chlorotic controls had only minor SPAD increases during the time of the experiment. In all chlorotic and green untreated leaves, the SPAD values of the distal part were always higher (22–41%) than that of the basal part (not shown). Also, some but not all leaves showed necrosis symptoms near the border of the untreated basal part (Figure 4C). Iron-sufficient control green leaves also had a SPAD value increase during the experimental period (approximately 45 and 30% for the basal and distal leaf parts, respectively; Figure 3B).

### Changes in leaf mineral concentrations in peach tree and sugar beet leaves with iron fertilization

In the case of field-grown peach trees, we used the experimental design and sampling protocol described in El-Jendoubi et al. (2011) to decrease field-induced variability as much as possible. The peach tree leaf mineral analysis data were first analyzed pooling data from the 3 years as replications (*n* = 11). Foliar Fe fertilization induced significant Fe concentration increases in the distal treated leaf part (Table 1). Also, the basal untreated part of fertilized leaves had slight Fe increases when compared to the basal part of untreated leaves, although the differences were only significant at *p* ≤ 0.10. The rest of mineral elements analyzed were not affected by Fe fertilization (Table 1). Calcium, N, and Mn concentrations tended to be different in the basal and distal parts (Ca and Mn being more abundant in the basal part and N being more abundant in the distal parts). The concentrations of P, K, Mg, Cu, and Zn were similar in all samples. An additional statistical analysis was carried out with the peach tree data, taking into account “years” and “trees” as factors and using contrasts. This analysis indicates that although some differences in the nutrient concentrations means could be detected over years (Appendix, Table A1), the only significant differences consistently found across years were for the Fe concentrations in Fe-fertilized vs. non-fertilized leaves, both when considering the distal (at *p* ≤ 0.01) and the basal parts (at *p* ≤ 0.05) (Appendix, Table A1). This is in agreement with the results obtained pooling samples from the 3 years of study. Also, in the additional analysis the interaction year × treatment was not significant (Appendix, Table A1) supporting the validity of the simpler approach used in Table 1.

**Table 1:** **Concentrations of macro- (N, P, Ca, Mg, and K; in % DW) and microelements (Fe, Mn, Cu, and Zn; in μg g^−1^ DW) in basal and distal parts of Fe-deficient peach tree leaves either not fertilized or 8 weeks after the first treatment with 2 mM FeSO_4_ and 0.1% surfactant**.

	**Basal leaf part**		**Distal leaf part**	
	**Not fertilized**	Fe-fertilized^*^	**Not fertilized**	**Fe-fertilized**
N	3.46 ± 0.18a	3.29 ± 0.23a	3.78 ± 0.20A	3.88 ± 0.23A
P	0.23 ± 0.01a	0.22 ± 0.01a	0.24 ± 0.01A	0.22 ± 0.02A
K	2.91 ± 0.10a	2.89 ± 0.07a	2.87 ± 0.08A	2.79 ± 0.09A
Ca	3.54 ± 0.33a	3.64 ± 0.33a	2.97 ± 0.22A	3.11 ± 0.22A
Mg	0.91 ± 0.03a	0.88 ± 0.33a	0.97 ± 0.04A	0.93 ± 0.03A
Fe	103.1 ± 7.3a	126.7 ± 16.9a	126.0 ± 15.3B	176.7 ± 16.4A
Mn	89.4 ± 6.1a	92.8 ± 5.4a	67.5 ± 3.8A	70.8 ± 6.4A
Cu	15.0 ± 2.4a	14.9 ± 2.3a	15.6 ± 2.0A	15.3 ± 1.7A
Zn	26.4 ± 1.5a	27.9 ± 1.6a	28.8 ± 1.5A	28.8 ± 1.8A

Data are means ± SE (n = 11 trees: 3 in 2009, 4 in 2010, and 4 in 2011; each sample was composed of 20 leaves, each from a different shoot from the same tree). Values followed by the same letter within the same row were not significantly different (Duncan test) at the p ≤ 0.05 level. Columns with data corresponding to Fe-fertilized leaves are labeled “Fe-fertilized” in case of the treated (distal) leaf area and “Fe-fertilized

* ” in case of the (basal) untreated area.

In sugar beet, leaf Fe concentrations in the distal treated part tended to increase upon fertilization (42%, Table 2; differences were only significant at *p* ≤ 0.10), although they were still lower than those found in leaves of green sufficient plants. Also, the basal untreated part of fertilized leaves had slight Fe increases (30%) when compared to the basal part of untreated leaves (again, differences were only significant at *p* ≤ 0.10). The sugar beet leaf macronutrient concentrations were not affected significantly by Fe fertilization (Table 2). Leaf N concentrations were similar in all samples, with the exception of the distal parts of Fe-sufficient plants, which were higher than those in the other treatments. Phosphorus concentrations were higher in the distal and basal leaf parts of the Fe-sufficient plants than in the other treatments, whereas no significant differences in K concentrations were found. In the case of Ca, the concentration was significantly higher in the Fe-fertilized and Fe-deficient leaves than in the Fe-sufficient controls. The concentrations of Ca and Mg tended to increase upon Fe fertilization (although only significantly at *p* ≤ 0.10), with the highest concentrations of both macronutrients being found in Fe-fertilized leaves. When considering the micronutrients, the concentrations of Mn tended to be higher in fertilized leaves than in the non-fertilized ones (differences significant at *p* ≤ 0.10), although values were not as high as those found in green Fe-sufficient plants; Mn concentrations were generally higher in the distal than in the basal part. In the case of Cu, concentrations decreased significantly with Fe fertilization in the distal leaf part when compared to the untreated leaves. Finally, Zn concentrations were not affected by Fe fertilization, and the concentrations in Fe-sufficient plants were the highest.

**Table 2:** **Concentrations of macro- (N, P, Ca, Mg, and K; in % DW) and microelements (Fe, Mn, Cu, and Zn; in μg g^−1^ DW) in basal and distal parts of Fe-deficient sugar beet leaves either not fertilized or 7 days after the first treatment with 2 mM FeSO_4_ and 0.1% surfactant**.

	**Basal leaf part**			**Distal leaf part**		
	**Not fertilized**	Fe-fertilized^*^	**Green, Fe-sufficient**	**Not fertilized**	**Fe-fertilized**	**Green, Fe-sufficient**
N	3.54 ± 0.32a	3.33 ± 0.16a	4.00 ± 0.42a	3.69 ± 0.12B	3.24 ± 0.16B	5.34 ± 0.13A
P	0.28 ± 0.03b	0.25 ± 0.03b	0.74 ± 0.07a	0.34 ± 0.04B	0.19 ± 0.02B	1.04 ± 0.28A
K	4.30 ± 0.17a	4.89 ± 0.28a	4.40 ± 0.06a	4.78 ± 0.41A	4.97 ± 0.43A	4.93 ± 0.31A
Ca	5.77 ± 0.16b	6.73 ± 0.36a	2.08 ± 0.08c	6.69 ± 0.54A	7.43 ± 0.55A	2.09 ± 0.08B
Mg	2.02 ± 0.21ab	2.42 ± 0.19a	1.81 ± 0.06b	2.13 ± 0.25A	2.75 ± 0.29A	2.16 ± 0.08A
Fe	104.3 ± 16.0a	135.3 ± 14.8a	151.1 ± 22.7a	145.8 ± 11.7B	207.0 ± 15.0AB	265.0 ± 48.4A
Mn	73.5 ± 13.4a	111.4 ± 18.2a	126.1 ± 20.4a	135.9 ± 23.0B	161.5 ± 8.0B	226.2 ± 13.4A
Cu	13.4 ± 1.9ab	10.6 ± 1.5b	17.7 ± 2.8a	19.0 ± 3.6B	9.6 ± 1.5C	34.4 ± 5.8A
Zn	27.6 ± 1.9b	18.5 ± 1.5b	61.5 ± 11.2a	23.6 ± 1.5AB	20.8 ± 1.5AB	110.4 ± 12.3A

The elemental concentrations of leaves from green, Fe-sufficient plants are also included for comparison. Data are means ± SE (n = 8 plants, 4 in each of two different batches; each sample was composed of two leaves from the same plant). Values followed by the same letter within the same row were not significantly different (Duncan test) at the p ≤ 0.05 level. Columns with data corresponding to Fe-fertilized leaves are labeled “Fe-fertilized” in case of the treated (distal) leaf area and “Fe-fertilized

* ” in case of the (basal) untreated area.

### Changes in the pigment concentrations in peach tree and sugar beet leaves with foliar iron fertilization

In peach trees, Fe fertilization increased significantly the concentrations per area of most pigments in the distal treated area of the leaf (Table 3; the only exception was zeaxanthin -Z-, data not shown). The largest increases were for Chl *b*, Chl *a*, and total Chl (2.6-, 2.4-, and 2.4-fold, respectively), and less marked in the case of the carotenoids neoxanthin, lutein, and β-carotene (83–88%). The total pool of violaxanthin (V) cycle pigments (violaxanthin + antheraxanthin + zeaxanthin; V+A+Z) increased by 54%, with V increasing by 74%. On the other hand, the concentration of photosynthetic pigments in the basal leaf part did not change significantly after Fe fertilization (Table 3).

**Table 3:** **Concentrations of photosynthetic pigments (in μmol m^−2^; Chl *a*, Chl *b*, neoxanthin, lutein, β-carotene, and V+A+Z) and Chl *a*/Chl *b* and Z+A/(V+A+Z) ratios in basal and distal parts of Fe-deficient peach tree leaves either not fertilized or 8 weeks after the first treatment with 2 mM FeSO_4_ and 0.1% surfactant (Fe-fertilized)**.

	**Basal leaf part**		**Distal leaf part**	
	**Not**	**Fe-**	**Not**	**Fe-**
	**fertilized**	fertilized^*^	**fertilized**	**fertilized**
Chl *a*	73.6 ± 4.0a	77.8 ± 6.5a	82.4 ± 3.4B	197.7 ± 7.7A
Chl *b*	20.1 ± 1.4a	25.0 ± 3.2a	24.5 ± 1.8B	63.3 ± 2.9A
Chl total	93.7 ± 5.2a	102.8 ± 8.5a	106.9 ± 4.9B	261.0 ± 10.5A
Neoxanthin	6.3 ± 0.3a	6.5 ± 0.5a	7.3 ± 0.3B	13.7 ± 0.7A
Lutein	14.7 ± 0.7a	15.5 ± 1.0a	17.2 ± 0.7B	31.6 ± 1.8A
β-carotene	14.7 ± 0.6a	15.0 ± 1.1a	17.1 ± 0.7B	31.3 ± 1.3A
(V+A+Z)	20.2 ± 1.1a	18.7 ± 1.6a	21.3 ± 1.2B	32.7 ± 2.3A
Chl *a*/Chl *b*	3.9 ± 0.1a	3.5 ± 0.2a	3.7 ± 0.1B	3.2 ± 0.1A
(Z+A)/(V+A+Z)	0.44 ± 0.04a	0.43 ± 0.05a	0.40 ± 0.04B	0.24 ± 0.04A

A second treatment was done at week 4. Data are means ± SE (n = 8 trees, 4 each in 2010 and 2011; each sample was composed of 4 leaf disks). Values followed by the same letter within the same row were not significantly different (Duncan test) at the p ≤ 0.05 level. Columns with data corresponding to Fe-fertilized leaves are labeled “Fe-fertilized” in case of the treated (distal) leaf area and “Fe-fertilized

* ” in case of the (basal) untreated area.

The Chl *a*/Chl *b* ratio in the distal treated part of Fe-deficient leaves decreased from 3.7 to 3.2 after fertilization (Table 3), whereas changes in the untreated basal part were not significant. Changes upon Fe fertilization were also found in the de-epoxidation state of the V+A+Z cycle, with the proportion of Z+A decreasing from 0.40 to 0.24 in the treated distal leaf parts. In the untreated basal part of Fe-fertilized plants the proportion of Z+A did not change upon fertilization.

In sugar beet, the foliar Fe treatment also led to increases in the concentration of photosynthetic pigments in the distal treated leaf area (Table 4). The increase was largest in the case of β-carotene, Chl *b*, and Chl *a*, (8.8-, 6.4-, and 6.0-fold, respectively), and less marked in the case of neoxanthin and lutein (4.8- and 4.6-fold, respectively). All pigment values found in the treated leaf areas after fertilization were still lower (in the range 44–76%) than those found in leaves of Fe-sufficient plants. Slight increases in pigments were also found in the basal untreated leaf parts (especially in the case of Chl *b*), but differences were not statistically significant at *p* ≤ 0.05. On the other hand, the pigment concentrations in the distal part of untreated sugar beet leaves were quite similar to those in the corresponding basal leaf parts.

**Table 4:** **Concentrations of photosynthetic pigments (in μmol m^−2^; Chl *a*, Chl *b*, neoxanthin, lutein, β-carotene, and V+A+Z) and Chl *a*/Chl *b* and Z+A/(V+A+Z) ratios in basal and distal parts of Fe-deficient sugar beet leaves either not fertilized or 7 days after the first treatment with 2 mM FeSO_4_ and 0.1% surfactant (Fe-fertilized)**.

	**Basal leaf part**			**Distal leaf part**		
	**Not fertilized**	Fe-fertilized^*^	**Green, Fe-sufficient**	**Not fertilized**	**Fe-fertilized**	**Green, Fe-sufficient**
Chl *a*	33.8 ± 2.0b	57.5 ± 12.0b	272.0 ± 46.0a	33.0 ± 1.4C	199.4 ± 28.1B	263.8 ± 27.8A
Chl *b*	6.6 ± 0.2b	20.4 ± 6.2b	82.52 ± 12.9a	8.5 ± 0.4C	54.4 ± 11.6B	83.5 ± 10.8A
Chl total	40.4 ± 2.2b	77.9 ± 16.7b	354.6 ± 58.8a	41.5 ± 1.0C	253.9 ± 39.3B	347.3 ± 37.4A
Neoxanthin	1.8 ± 0.2b	2.1 ± 0.3b	14.1 ± 1.8a	1.4 ± 0.3C	6.7 ± 1.4B	15.3 ± 3.7A
Lutein	7.1 ± 1.0b	9.5 ± 6.6b	44.6 ± 8.4a	5.5 ± 0.8C	25.5 ± 0.6B	54.0 ± 8.8A
β-carotene	2.4 ± 0.8b	5.3 ± 0.6b	30.6 ± 5.6a	2.4 ± 1.2C	21.1 ± 3.8B	41.1 ± 9.4A
(V+A+Z)	10.4 ± 1.3b	10.2 ± 2.0b	22.5 ± 4.1a	8.2 ± 1.3C	14.5 ± 1.7B	27.9 ± 5.9A
Chl *a*/Chl *b*	5.1 ± 0.2a	3.2 ± 0.6b	3.3 ± 0.8b	3.9 ± 0.4A	3.8 ± 0.3A	3.2 ± 0.2A
(Z+A)/(V+A+Z)	0.77 ± 0.04a	0.57 ± 0.14b	0.02 ± 0.01c	0.78 ± 0.5A	0.16 ± 0.08B	0.04 ± 0.01A

The pigment concentrations of leaves from Fe-sufficient plants are also shown for comparison. Data are means ± SE (n = 4 plants; each sample was composed of two leaves from the same plant). Values followed by the same letter within the same row were not significantly different (Duncan test) at the p ≤ 0.05 level. Columns with data corresponding to Fe-fertilized leaves are labeled “Fe-fertilized” in case of the treated (distal) leaf area and “Fe-fertilized

* ” in case of the (basal) untreated area.

On the other hand, the Chl *a*/Chl *b* ratio did not change after the Fe treatment in the distal treated leaf parts, but showed decreases in the basal part (from 5.1 to 3.2) (Table 4). The (Z+A)/(V+A+Z) ratio decreased markedly in the distal treated leaf part after the Fe treatment, and also decreased in the untreated basal part, although to a lower extent. The highest (Z+A)/(V+A+Z) ratio was found in chlorotic leaves and the lowest in green leaves.

### Localization of iron labile pools by perls-DAB stain in transversal sections of peach tree leaves as affected by iron fertilization

**Figure 5:**
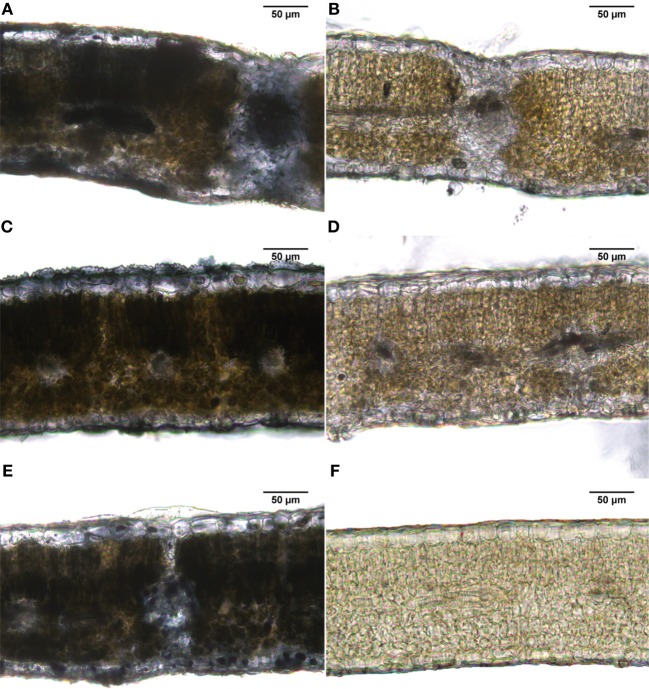
**Iron staining (Perls-DAB) in leaf peach tree transversal sections. (A)** Fe-sufficient control; **(B)** Fe-deficient chlorotic; **(C)** distal treated leaf part (2 mM FeSO_4_ with 0.1% surfactant); **(D)** basal untreated leaf part in the same leaves used for **(C)**; **(E)** leaves of a soil Fe-fertilized tree (Fe(III)-EDDHA -Sequestrene-, 50 g per tree); and **(F)** negative control.

The Perls-DAB staining method indicates the localization of labile Fe pools with a dark color. In control, foliar Fe-fertilized and soil Fe-fertilized peach tree leaves, labile Fe pools were located in most of the leaf cross-section, with a lower intensity in the upper epidermal layer (Figures 5A,C,E, respectively). In Fe-deficient and the basal untreated part of Fe-fertilized leaves, some Fe labile pools were found in vascular tissues and to a minor extent in the parenchymal areas (Figures 5B,D, respectively). Figure 5F shows a negative control (without DAB) of a Fe-deficient leaf.

### Structure and semi-quantitative relative distribution of total Fe (LT SEM-EDX) in cryo-fractured transversal sections of peach tree leaves as affected by iron fertilization

**Figure 6:**
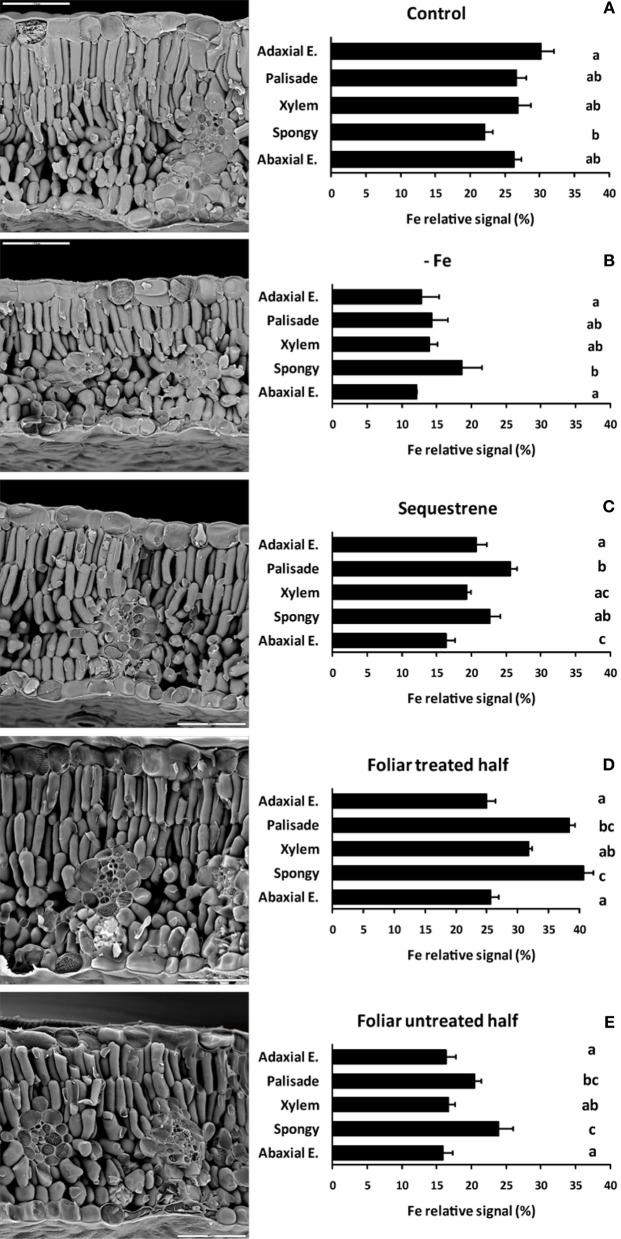
**LT-SEM micrographs (left panels) and semi-quantitative EDX analysis (spot mode, right panels) of transversal sections obtained by cryo-fracture from peach tree leaves. (A)** Fe-sufficient control; **(B)** Fe-deficient chlorotic; **(C)** soil Fe-fertilized (Fe(III)-EDDHA –Sequestrene-, 50 g per tree); **(D)** distal Fe-treated leaf part (2 mM FeSO_4_ with 0.1% surfactant); and **(E)** basal untreated leaf part in the same leaves used for **(D)**. Relative Fe signals are means (± SE). Significant differences among plant tissues are indicated by different letters (*p* ≤ 0.05; *n* = 8). Bars in the images are 50 μm.

Leaf tissue structural information of the different layers of cryo-fractured peach tree leaves, including adaxial epidermis, palisade parenchyma, xylem vessels, spongy parenchyma, and abaxial epidermis, was obtained using LT SEM. Chlorotic leaves had a more compact mesophyll tissue (Figure 6B, left panel) when compared to the green controls (Figures 6A,C,D, left panels). The distribution of the relative Fe signals (semi-quantitative analysis) in the leaf-cross sections obtained by EDX analysis is also shown in Figure 6 (right panels). Iron signals were markedly more intense in leaf sections of control and Fe-fertilized samples (Figures 6A,C,D) than in those of Fe-deficient and untreated leaf areas (Figures 6B,E). Also, the relative Fe signal in the untreated area of the half treated leaves (Figure 6E) was more intense than in the Fe-deficient leaves (Figure 6B). In Fe-deficient leaves, the relative Fe signal was more intense in the spongy parenchyma in comparison with the rest of leaf tissues (Figure 6B), whereas in control leaves the relative Fe signal was more intense in the adaxial epidermal layer and somewhat lower in spongy parenchyma (Figure 6A). In the distal sections of Fe-fertilized leaves, more intense Fe signals were present in palisade and spongy parenchyma and to a lower extent in the xylem area; this occurred both after soil (Figure 6C) and foliar Fe-fertilization (Figure 6D). Also, some increases in the relative intensity of the Fe signal occurred in the palisade and spongy parenchyma in the basal untreated leaf part (Figure 6E) when compared to the Fe-deficient control (Figure 6B).

### Quantitative Fe distribution in transversal sections of peach tree leaves using STIM-μPIXE as affected by iron fertilization

**Figure 7:**
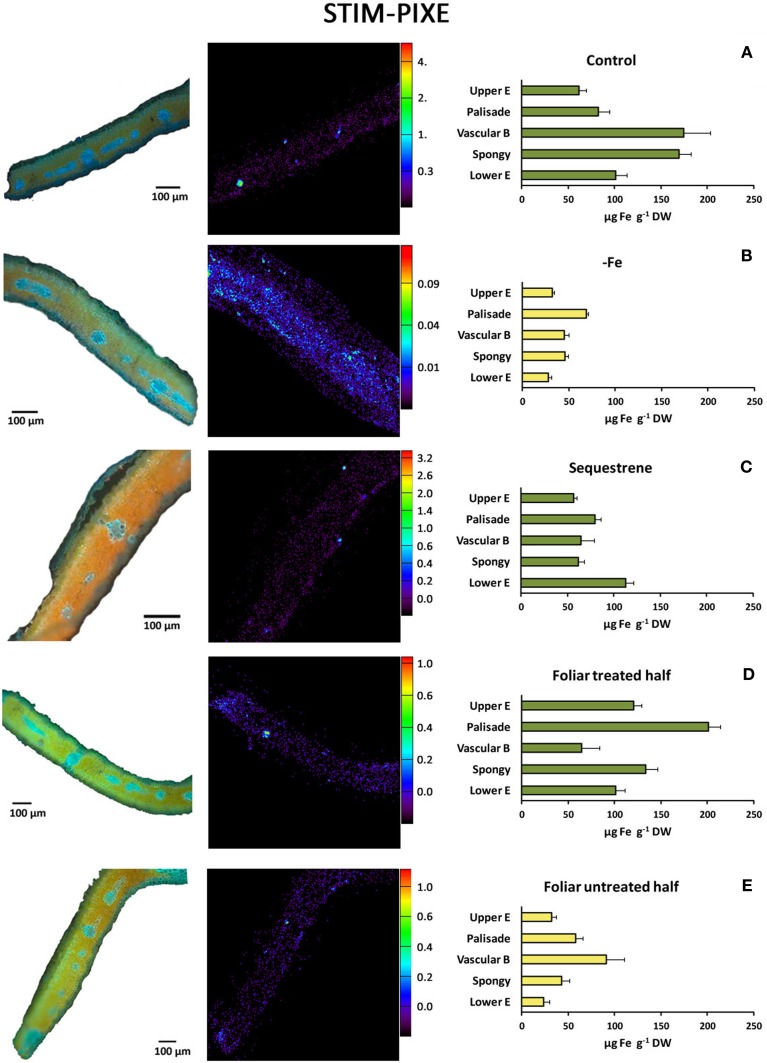
**STIM μ-PIXE mapping and quantitative analysis of Fe (right panels) of selected areas from transversal sections of peach tree leaves. (A)** Fe-sufficient control; **(B)** Fe-deficient chlorotic; **(C)** soil Fe-fertilized (Fe(III)-EDDHA -Sequestrene-, 50 g per tree); **(D)** distal Fe-treated leaf part (2 mM FeSO_4_ with 0.1% surfactant); and **(E)** basal untreated leaf part in the same leaves used for **(D)**. Signals are means in μg Fe g^−1^ DW (± SE).

This quantitative method showed higher levels of Fe in green leaves than in chlorotic ones (Figure 7). Also, the Fe signal in the untreated area of the half treated leaves was more intense than that in the Fe-deficient leaves. These data generally agree with the leaf Fe concentrations shown in Table 1. In Fe-deficient leaves, the Fe signal was more intense in the palisade parenchyma in comparison with the rest of leaf tissues (Figure 7B), whereas in control leaves the Fe signal was more intense in the vascular tissue and the spongy parenchyma (Figure 7A). In the distal sections of Fe-foliar fertilized leaves, more intense Fe signals were present in palisade, spongy parenchyma and upper epidermis and to a lower extent in the vascular area (Figure 7D), whereas in the soil-fertilized ones the highest concentration was in the lower epidermis (Figure 7C). Also, some increases in the intensity of the Fe signal occurred in the vascular area in the basal untreated leaf part (Figure 7E).

### Changes in CHL fluorescence in peach tree leaves with iron fertilization

**Figure 8:**
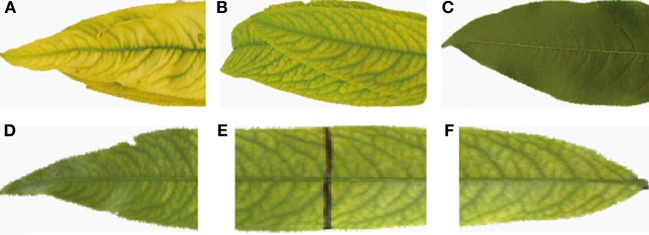
**Peach tree leaves used for the Chl fluorescence measurements. (A)** Severely chlorotic leaf, with a very advanced chlorosis, taken from the distal part of the shoot; **(B)** Fe-deficient leaf taken at the 4^th^–5^th^ position in the shoot, one week after treatment by dipping the distal half of the leaf in a solution containing 2 mM FeSO_4_ and 0.1% surfactant; **(C)** Positive control: Fe-sufficient leaves taken in the same position in the shoot but from a Fe-sufficient tree; **(D)** distal part of an Fe-treated leaf; **(E)** middle part of an Fe-treated leaf, showing the black line delimiting the treatment area; and **(F)** basal part of an Fe-treated leaf.

**Figure 9:**
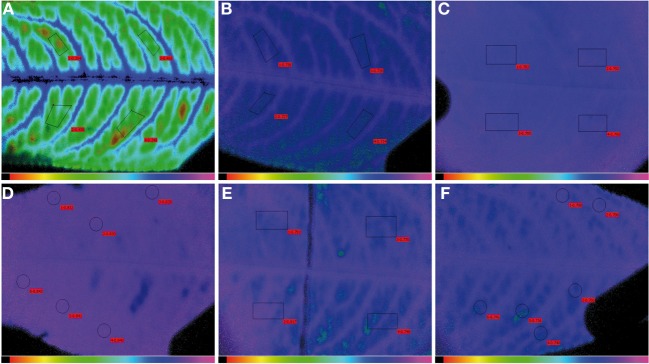
**Images showing the difference in dark-adapted, maximum potential PSII efficiency (F_V_/F_M_) in peach tree leaves. (A)** A severely Fe-deficient leaf, having 61 μmol Chl m^−2^; **(B)** an Fe-deficient leaf, having 95 μmol Chl m^−2^; **(C)** an Fe-sufficient leaf having 350 μmol Chl m^−2^; **(D)** distal part of an Fe-treated leaf; **(E)**, middle part of an Fe-treated leaf, showing the black line delimiting the treatment area; and **(F)** basal part of an Fe-treated leaf. Areas measured for data shown in Table 5 are tagged in red.

Chl fluorescence was imaged and measured in severely and moderately Fe-deficient, Fe-sufficient and Fe-fertilized peach tree leaves 1 week after the first foliar Fe application (Figure 8). Fluorescence images are shown in false color code in Figure 9, and quantitative values found in the leaf areas tagged in red in Figure 9 are shown in Table 5.

**Table 5:** **Chl fluorescence parameters (F_V_/F_M_, Φ_PSII_, qP, and NPQ) in severely and moderately Fe-deficient, distal treated and basal untreated areas of fertilized leaves, and Fe-sufficient peach tree leaves**.

	**Severe Fe**	**Moderate Fe**	**Moderate deficiency**				**Green Fe-sufficient**
	**deficiency**	**deficiency**					
	**Not fertilized**	**Not fertilized**	**Fe-fertilized**				
			**More distal part**	**Distal part**	**Basal part**	**More basal part**	
F_V_/F_M_	0.61 ± 0.05d	0.74 ± 0.01bc	0.82 ± 0.01a	0.80 ± 0.01ab	0.77 ± 0.01abc	0.71 ± 0.01c	0.80 ± 0.01ab
Φ_PSII_	0.38 ± 0.04c	0.51 ± 0.01ab	0.55 ± 0.01a	0.55 ± 0.00a	0.54 ± 0.01a	0.48 ± 0.02b	0.49 ± 0.02ab
qP	0.80 ± 0.01a	0.78 ± 0.02ab	0.73 ± 0.01bc	0.76 ± 0.01abc	0.79 ± 0.01b	0.74 ± 0.02bc	0.71 ± 0.02c
NPQ	0.16 ± 0.01b	0.13 ± 0.01b	0.14 ± 0.01b	0.15 ± 0.01b	0.14 ± 0.01b	0.09 ± 0.01c	0.20 ± 0.02a

Data are means ± SE (n = 12, 4–6 areas of interest in each of 3 leaves). Data followed by the same letter within the same row are not significantly different (Duncan test) at the p ≤ 0.05 level.

Typical F_V_/F_M_ images for the different treatments are shown in Figure 9, and the numerical F_V_/F_M_ values are shown in Table 5. The image in Figure 9A is an example from a severely Fe-deficient leaf, which had very low Chl concentrations and a low F_V_/F_M_ ratio. Slight differences in the F_V_/F_M_ images from Fe-deficient and Fe-sufficient controls can be observed in Figures 9B,C, respectively, although the measured difference in F_V_/F_M_ was only statistically different at *p* ≤ 0.10 (Table 5). In all Fe-deficient leaves, areas close to the veins had a higher F_V_/F_M_ ratio than the corresponding interveinal areas (Figures 9A,B). One week after the treatment, the more distal areas showed F_V_/F_M_ ratios similar to those of the Fe-sufficient controls (Figures 9D,C, respectively; Table 5). The F_V_/F_M_ imaging suggests that in this distal area ratios may decrease slightly when approaching the treatment line border (Figure 9E, left side), although F_V_/F_M_ values were not significantly different (Table 5). In the basal untreated part, F_V_/F_M_ ratios were lower than those of the treated part and similar to those of chlorotic leaves (Table 5).

Concerning Φ_PSII_, it was lower in the severely Fe-deficient leaves than in moderately Fe-deficient and Fe-sufficient ones (Table 5). Upon Fe resupply, the distal treated parts showed an increase of Φ_PSII_ values when compared to the basal untreated part; an increase in this parameter was also observed in the basal part close to the treatment border. In the case of qP and NPQ, values were higher and lower, respectively, in the Fe-deficient leaves than in the Fe-sufficient controls. In the treated leaves, qP decreased only in the more distal area, whereas NPQ did not show significant changes (Table 5).

## Discussion

Results show that treatments with Fe-sulfate on chlorotic, Fe-deficient leaves were effective at the site of application, both in peach trees grown in the field and in sugar beet grown in hydroponics. Application of 2 mM FeSO_4_ to the distal parts of peach tree and sugar beet leaves caused similar increases in the Fe concentrations in the treated parts (41–42%). Iron entered most of the leaf tissues, with the increases being large in palisade and spongy parenchyma and also present in vascular tissues, as indicated by semi-quantitative SEM-EDX and quantitative STIM-μPIXE. Furthermore, the Perls stain results indicate that Fe fertilized leaves have labile Fe pools across the leaf width.

The entrance of Fe in the leaf treated area resulted in rapid and significant re-greening, confirming data found in previous studies with peach trees (Fernández et al., 2006, 2008). Increases in SPAD values were already significant at the first sampling dates after the treatment, 1 d in sugar beet and 1 week in peach trees, and this re-greening kinetics is also in good agreement with previous data for sugar beet (Larbi et al., 2004) and peach trees (El-Jendoubi et al., 2011). At the end of the experiment, SPAD values had increased, when compared to the initial leaf SPAD values, by less than 2-fold in peach and 9-fold in sugar beet. In previous studies with peach and pear trees the Chl increases after foliar Fe fertilization were 2- (Fernández et al., 2006) and 3-fold (Álvarez-Fernández et al., 2004), respectively. Regarding the relative increases in photosynthetic pigments, the increases were in the order Chl *b* > Chl *a* > neoxanthin > lutein > β-carotene > V+A+Z in peach tree leaves, and β-carotene > Chl *b* > Chl *a* > neoxanthin > lutein > V+A+Z in sugar beet leaves. These changes were accompanied by decreases in the (Z+A)/(V+A+Z) ratio in both species, as well as by small increases in F_V_/F_M_ in peach tree leaves. Iron deficiency has been shown to induce decreases in F_V_/F_M_ and Φ_PSII_ in sugar beet, peach and pear (Nedunchezhian et al., 1997; Abadía et al., 1999; Morales et al., 2000), and similar changes in photosynthetic pigments and Chl fluorescence after Fe-resupply to the nutrient solution were reported to occur in sugar beet (Larbi et al., 2004).

The effects of foliar Fe treatments on the basal, untreated parts of chlorotic Fe-deficient leaves were very limited. Application of FeSO_4_ to the distal parts of leaves caused small increases in the Fe concentrations in the untreated parts (23–30% in peach trees and sugar beet, respectively), which were statistically significant only at *p* ≤ 0.10. The use of semi-quantitative SEM-EDX suggested that some Fe did enter the spongy and palisade parenchyma, whereas the Perls stain and quantitative STIM-PIXE also suggested a slight increase of labile Fe forms in some vascular areas. This small Fe increase is unlikely to result from surface mass flow movement of Fe compounds at the moment of application, because all treated leaf surfaces dried within a few minutes. The measurable leaf entrance of Fe, however, resulted in only very minor leaf re-greening, given that the bulk concentration of photosynthetic pigments in the basal untreated part did not change, although a decrease in the (Z+A)/(V+A+Z) and Chl *a*/Chl *b* ratios was found when compared to the untreated controls. The lack of effect of the Fe concentration increases on the pigment concentrations suggests that most of the new Fe coming from the fertilizer in the untreated leaf areas was in forms and/or localizations that cannot be used for chloroplast Fe resupply. Although it could be argued that the amount of Fe applied was only sufficient for re-greening the treated part, and that more Fe could be necessary to produce full recovery of the untreated part, the relatively high Fe concentrations found in the treated leaf areas (177 and 207 μg g^−1^ DW in peach and sugar beet, respectively; Tables 1, 2) point out to difficulties in Fe remobilization in the Fe-treated leaves.

The lack of significant re-greening of the untreated leaf parts after Fe sulfate fertilization of chlorotic, Fe-deficient leaves found in this study are in line with results in previous studies in peach (Fernández et al., 2008) and grapevine (Yunta et al., 2013). This is in contrast with results obtained in cereal crops such as wheat and rice, which are usually carried out in the absence of leaf chlorosis, where applied Fe re-translocates efficiently to other plant organs including grains (Cakmak et al., 2010; Zhang et al., 2010; Aciksoz et al., 2011; Wei et al., 2012; He et al., 2013). The reasons behind this lack of efficiency could reside in different biochemical changes induced by Fe-chlorosis, including increases in the pH of the xylem sap and leaf apoplast, as well as in the carboxylate concentrations in these compartments, which may decrease the efficiency of the Fe-uptake mechanisms in leaf mesophyll cells, and especially the Fe(III)-chelate reductase plasma membrane enzyme [(González-Vallejo et al., 2000); see review by Abadía et al. (2002) and references herein]. These constraints are unlikely to occur in leaves without chlorosis symptoms. On the other hand, delivering Fe foliar treatments to non-graminaceous plants that do not show chlorosis symptoms may compromise root Fe uptake with unknown effects on long term plant Fe status.

Previous results indicating that Fe foliar fertilization could lead to a switch in nutrient composition in peach tree leaves, from a high (K–N–P)/low (Ca–Mg) to a high (Ca–Mg)/low (K–N–P) state (Fernández et al., 2008) were not confirmed in the present study. The origin of this discrepancy is unclear, although in the cited study (Fernández et al., 2008) nutrient concentrations used for the calculations were the average of those found with several foliar Fe-treatments, using not only FeSO_4_ but also a number of other Fe-containing formulations. This issue should be clarified in further studies.

The changes in Chl imaging fluorescence parameters found with Fe deficiency and Fe-resupply in this study were less marked than those found for whole leaf Chl fluorescence parameters in previous studies (Morales et al., 1994, 2000; Nedunchezhian et al., 1997; Abadía et al., 1999).This could be assigned to the differences in the Chl fluorescence devices used, since there are examples in the literature that using different devices leads to significant differences in Chl fluorescence parameters (Peguero-Pina et al., 2009). For instance, with the PAM-2000 it is possible to use a protocol that includes a far-red (FR) pre-illumination after leaf dark-adaptation, and this leads to increases in the F_V_/F_M_ values of Fe-deficient leaves (Belkhodja et al., 1998a). Unfortunately, with the imaging-PAM it is not currently possible to use FR pre-illumination, making comparisons with the PAM-2000 difficult. In any case, changes found in most parameters with Fe-deficiency and resupply had a consistent trend, with parameters in distal Fe-treated areas approaching values found in the controls. The same occurred in untreated basal areas close to the application, although in this case to a lesser extent. The case of qP merits a brief commentary, since although there was no significant difference between the qP values in both parts of the treated leaves, values were always high; the highest value was found in severely chlorotic peach leaves (0.80), and the lowest one in the green leaves (0.71). A similar result was obtained in an earlier work with sugar beet, and it was proposed that an alternative PSII electron acceptor may consume electrons from Q_A_, the primary quinone electron acceptor in PSII, and/or the plastoquinone pool, maintaining oxidized the PSII acceptor side (Morales et al., 1998).

In summary, the application of a foliar fertilizer containing FeSO_4_ to Fe-deficient, chlorotic leaves was effective only at the leaf treated surface, both in peach trees grown in the field and sugar beet grown in hydroponics. Iron was thoroughly incorporated in the leaves and the re-greening was very marked. The effects of the foliar fertilizer, however, were very minor outside the leaf surface treated. This indicates that the major focus of new studies on foliar Fe fertilization should be put on internal Fe transport within the leaf, and new formulations should be aimed to extend the reach of the Fe fertilizers beyond the treated area. Whereas basic Fe transport mechanisms in the plant have been recently unraveled [see reviews by Abadía et al. (2011) and Samira et al. (2013)], including xylem Fe transport as Fe-carboxylate complexes (Rellán-Álvarez et al., 2010), Fe reduction by mesophyll cells (González-Vallejo et al., 2000; Larbi et al., 2001) and nicotianamine-dependent phloem Fe unloading (Schuler et al., 2012), very little is known on the Fe transport mechanisms occurring after foliar Fe fertilization. New knowledge on the Fe mobilization pathways in the Fe-fertilized leaves will be necessary to improve fertilization efficiency.

### Conflict of interest statement

The authors declare that the research was conducted in the absence of any commercial or financial relationships that could be construed as a potential conflict of interest.

## Appendix

**Table A1:** **Statistical analysis using “years” as a fixed factor with “trees” nested into years of the same Fe data used in Table 1**.

	**2009**		**2010**	**2011**	
**BASAL LEAF PART**					
Not fertilized	137.1 ± 8.0		85.1 ± 3.2	95.7 ± 3.1	
Fe-fertilized^*^	210.6 ± 3.4		95.6 ± 8.8	94.8 ± 8.5	
**DISTAL LEAF PART**					
Not fertilized	192.9 ± 12.9		102.8 ± 18.7	99.1 ± 4.2	
Fe-fertilized	253.2 ± 13.7		154.7 ± 13.0	141.4 ± 6.6	
**Source**	**DF**	**Type III SS**	**Mean square**	***F* value**	**Pr > F**
Year	2	70415.43390	35207.71695	68.03	^**^
Tree (year)	8	3436.36089	429.54511	0.83	NS
Treatment	3	33684.89635	11228.29878	21.70	^**^
Year × treatment	6	6602.81550	1100.46925	2.13	NS
Error	24	12420.0821	517.5034		
**Contrast**	**DF**	**Contrast SS**	**Mean square**	***F* value**	**Pr > F**
Distal Fe-fertilized vs. distal-not fertilized	1	14147.22821	14147.22821	22.31	<0.0001
Basal Fe-fertilized vs. basal-not fertilized	1	3047.53548	3047.53548	4.81	0.0363

Specific contrasts were carried out to compare the Fe-fertilized vs. the non-fertilized basal parts and the Fe-fertilized vs. the non-fertilized distal parts. Rows with data corresponding to Fe-fertilized leaves are labeled “Fe-fertilized” in case of the treated (distal) leaf area and “Fe-fertilized

* ” in case of the (basal) untreated area. NS and

** indicate non-significant and significant difference at the *p* ≤ *0.01* level, respectively.
